# Peer Support for Patients With Heart Failure: A Systematic Review and Meta-Analysis

**DOI:** 10.7759/cureus.46751

**Published:** 2023-10-09

**Authors:** Shuri Nakao, Tomohiko Kamo, Hidehiro Someko, Masatsugu Okamura, Yasushi Tsujimoto, Hirofumi Ogihara, Shinya Sato, Sokichi Maniwa

**Affiliations:** 1 Division of Rehabilitation Medicine, Shimane University Hospital, Izumo, JPN; 2 Department of Physical Therapy, Faculty of Rehabilitation, Gunma Paz University, Takasaki, JPN; 3 Department of Systematic Reviewers, Scientific Research Works Peer Support Group (SRWS-PSG), Osaka, JPN; 4 Department of General Internal Medicine, Asahi General Hospital, Asahi, JPN; 5 Berlin Institute of Health Center for Regenerative Therapies (BCRT), Charité – Universitätsmedizin Berlin, Berlin, DEU; 6 Department of Rehabilitation Medicine, School of Medicine, Yokohama City University, Yokohama, JPN; 7 Department of Internal and Family Medicine, Oku Medical Clinic, Osaka, JPN; 8 Department of Health Promotion and Human Behavior, Kyoto University Graduate School of Medicine/School of Public Health, Kyoto University, Kyoto, JPN; 9 Division of Physical Therapy, Department of Rehabilitation, Faculty of Health Sciences, Nagano University of Health and Medicine, Nagano, JPN; 10 Department of Rehabilitation Medicine, Faculty of Medicine, Shimane University Hospital, Izumo, JPN

**Keywords:** systematic review, mortality, peer support, social support, heart failure

## Abstract

Peer support, which is given by people with similar life experiences and experiential knowledge, has been shown to be effective for patients with diabetes and mental illness. However, the impact of such peer support on patients coping with heart failure remains indeterminate. The objective of this systematic review and meta-analysis is to scrutinize the potential benefits of peer support for patients with heart failure. We included randomized controlled trials (RCTs) evaluating the effectiveness of peer support for patients with heart failure in contrast to those without peer support. We searched the Cochrane Central Register of Controlled Trials, MEDLINE, Embase, WHO International Clinical Trials Registry Platform, and ClinicalTrials.gov until October 2022. We pooled the data on mortality, readmission rate, and quality of life (QoL) as primary outcomes. The certainty of evidence was evaluated by the grading of recommendations assessment, development, and evaluation (GRADE) approach. We included three studies with 390 patients with heart failure. Peer support may have resulted in a slight increase in mortality (risk ratio (RR)=1.16, 95% confidence interval (CI)=0.61-2.21; low certainty of the evidence) and in a reduction in the readmission rate (RR=0.93, 95% CI=0.74-1.17; low certainty of the evidence). The evidence was very uncertain about the effect of peer support on QoL (standardized mean difference 2.03 higher in the intervention group, 95% CI=1.79 lower to 5.84 higher; very low certainty of the evidence). Despite that the certainty is low or very low, the extant data available evidence suggests that peer support may not yield substantial improvements in critical outcomes for patients with heart failure. Consequently, endorsing peer support for patients with heart failure currently seems unjustifiable.

## Introduction and background

Heart failure represents a complex syndrome presenting significant clinical and societal challenges, including escalated morbidity, mortality, and substantial strain on global healthcare systems [1]. By the year 2030, projections estimate that eight million individuals in the United States, with two million of these demographics being aged 80 and above [2]. Heart failure is also a prominent driver of hospitalization in the United States for the elderly population aged 65 years and older, with persistently elevated rates of rehospitalization [3]. For the therapeutic management of patients with heart failure with reduced ejection fraction (EF) (HFrEF), an integrated treatment approach comprising medication, exercise therapy, and multidisciplinary disease management is advocated to mitigate cardiovascular mortality, reduce rehospitalization, and enhance the quality of life (QoL) [4]. Several strategies, encompassing healthcare professional-led patient education and group education, have been proposed for multidisciplinary disease management in patients with heart failure, yet none have demonstrated sufficient evidence to substantiate a concrete clinical benefit [5].

Peer support, a constituent of disease management strategies, is defined as the process of providing and receiving non-clinical aid through an empathetic relationship to surmount severe mental, psychological, or addiction challenges, thereby facilitating long-term recovery [6-7]. Peer support was originally introduced in the field of mental health [8]. It has been reported that peer support can enhance hemoglobin A1c (HbA1c) control in patients with diabetes mellitus (DM) [9]. Heart failure, as well as DM, have aspects that require self-management, and peer support is a realistic approach to improve this self-management [10]. The implementation of peer support as a method for disease management in patients with heart failure has been introduced, yet adherence rates remain lackluster, and the efficacy of peer support is not definitively known [11]. Some studies infer that peer support may not be beneficial. For instance, it does not improve survival rates in patients with breast cancer [12]. Furthermore, peer support used by patients with depression via social networking services (SNS) may exacerbate their depressive tendencies [13].

To our knowledge, no report has summarized the evidence for peer support in patients with heart failure. A prior systematic review unveiled salutary effects of peer support for individuals afflicted with heart disease, encapsulating heightened self-efficacy, augmented physical activity, ameliorated pain, and diminished frequency of emergency room visits [14]. Nevertheless, significant outcomes, such as mortality and rehospitalization rates among patients with heart failure, were not included in the evaluation. After this review, a randomized controlled trial (RCT) focusing on peer support in patients with heart failure has been published [15-16]. The objective of the present study is to assess the effectiveness of peer support in patients with heart failure.

## Review

Materials and methods

We followed the Cochrane Handbook [17] and conducted a systematic review of the relevant literature. This systematic review protocol was registered with the open science framework (OSF) (https://osf.io/uxgam/), and in concordance with the 2020 PRISMA checklist (Supplementary Table 1), this systematic review deemed to align with PRISMA compliant.

We included randomized controlled trials (RCTs) that assess individual randomization or cluster randomization. We did not impose limits on countries or languages. Our analysis encompassed all manuscripts, both published and unpublished articles, in addition to the abstract of conference abstracts and correspondence. Studies were not precluded based on the observational duration or year of publication. The participants in question were patients who had received a diagnosis of heart failure and were treated in either an inpatient or outpatient medical setting. Patients exhibiting severe cognitive impairment or psychiatric disorders, with higher brain dysfunction after stroke or head injury, actively using narcotics or alcohol, serious visual or hearing impairment, and those actively undergoing cancer treatment were excluded from the study. The intervention method under consideration was peer support among patients, defined as a symbiotic relationship of mutual assistance between individuals sharing analogous life experiences [6]. Any mode of communication was deemed acceptable for peer support, including the exchange of concerns, queries, action plans, and progress concerning heart failure self-management. The frequency of peer support interaction was mandated to be at least once a week, with a minimum duration of one month. The control group comprised individuals receiving usual care and no peer support among patients. The control group included patients who did not partake in peer support and received healthcare professional-led heart failure coaching, participation in remote heart failure monitoring using apps or other means, and group heart failure coaching.

The primary outcomes were mortality, readmission rate, and quality of life (QoL). The mortality and readmission rates were included within one year following the intervention [15]. The QoL and self-efficacy [18] were included within six months following the intervention [15,19]. We allowed all validated QoL assessment scales, such as the 36-item short-form health survey questionnaire (SF-36) [20] and the Minnesota Living with Heart Failure Questionnaire (MHFQL) [21]. Secondary outcomes were the time to readmission, self-efficacy, and all adverse events. Self-efficacy was included within six months following the intervention. We allowed all validated self-efficacy assessment scales, such as self-care of heart failure index (SCHFI) [22].

We conducted a thorough search of MEDLINE (PubMed), the Cochrane Central Register of Controlled Trials (Cochrane Library), EMBASE (Dialog), the World Health Organization International Clinical Trials Platform Search Portal (ICTRP), and Clinical Trials.gov. Two independent reviewers checked the studies using the title and abstract. All extracted studies by the two reviewers underwent a comprehensive full-text review. The full text was subsequently utilized to ascertain the eligibility by each reviewer. In instances where only an abstract was available and the eligibility remained ambiguous, we contacted the original author. Discrepancies between the two reviewers were discussed and resolved, with a third reviewer consulted if necessary.

We extracted data on study characteristics and outcomes, including year of publication, author, and age. We extracted the difference between the means of the data for each outcome after the intervention period. However, if the average was not available, the difference between the average of the changed values and their standard deviation was extracted. We used the relative risk ratios (RRs) and the 95% confidence intervals (CI) as an effect measure for the following binary variables: readmission and mortality rate. We used the mean difference (MD), where appropriate, standardized MD (SMD), and the 95% CI as an effect measure for the following continuous variables: QoL and self-efficacy. We summarized adverse events based on the definition in the original article, but we did not perform the meta-analysis. For the integration of means and standard deviations of continuous variables, we followed the method of the Cochrane Handbook [17]. We endeavored to extract the intention-to-treat (ITT) analysis for all data as comprehensively as possible. Two independent reviewers carried out data extraction, resolving any arising discrepancies through discussion, or, if necessary, with a third reviewer. If needed, original authors were contacted for missing data. A pre-verified form was employed for the data extraction process. Two reviewers assessed the risk of bias independently using the risk-of-bias tool 2 [23], with disagreements resolved through discussion or consultation with a third reviewer if required.

We performed a meta-analysis with a random effects model using Review Manager software (RevMan 5.4.2). For continuous outcomes, we pooled MD with 95% CI. The SMD was applied if outcome measures differed across studies. For binary outcomes, we pooled RR with 95% CI. We used forest plots to visually evaluate the heterogeneity of the studies included. Consequently, we calculated I2 statistics (I2 values of 0%-40%: might not be important; 30%-60%: may represent moderate heterogeneity; 50%-90%: may represent substantial heterogeneity; 75%-100% considerable heterogeneity). Cochrane Chi2test (Q-test) was performed for I2 statistics, with a p-value less than 0.10 was denoted as statistically significant.

We made a summary of finding (SoF) tables based on the recommendations of the Cochrane Handbook [17]. SOF tables were created for the following outcomes: mortality, readmission rate, QoL, and self-efficacy. Each SoF included the quality of evidence based on the grading of recommendations assessment, development, and evaluation (GRADE) approach [17,24]. The certainty of the evidence was independently assessed by two researchers, with disagreements resolved through discussion, or the intervention of a third reviewer if needed.

Owing to data limitations, certain pre-specified analyses, such as subgroup analyses with age, EF, and peer support intervention methods, as well as sensitivity analyses, excluding studies utilizing imputation statistics and those with high overall RoB, were not executed. We had planned the time to readmission to the secondary outcome, but this was not implemented due to an inability to extract pertinent data from the included studies.

Results

**Figure 1:**
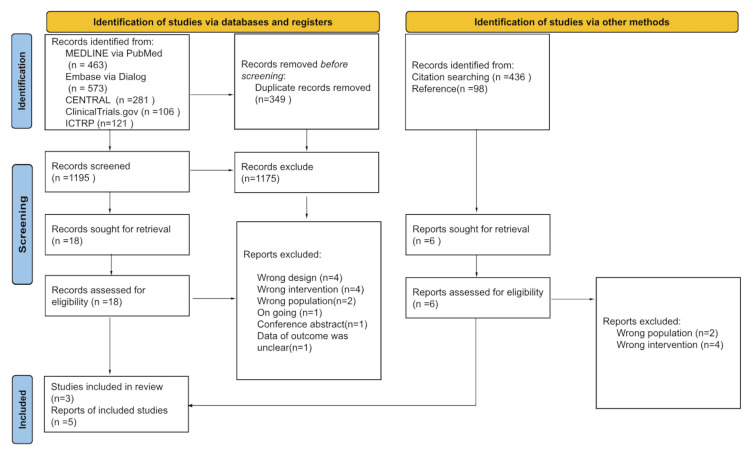
PRISMA flow chart

We exhaustively searched for relevant articles in October 2022. A total of 1195 articles were screened, and 24 full texts were assessed for eligibility (Figure 1).

A total of 19 studies were subsequently excluded following full-text screening. As a result, three studies comprising 390 patients with heart failure were definitively included. The characteristics of the included studies are presented in Table 1.

**Table 1:** Characteristics of the included studies RCT: randomized controlled trial, I: intervention group, C: control group, MLHFQ: Minnesota Living with Heart Failure Questionnaire, SCHFI: self-care heart failure index

Study	Country	Study design	Study duration	Study follow-up	Age (years) (I/C)	Sample size (I/C)	Patient group characteristics	Intervention method	Outcome
Heisler et al. 2013 [15]	USA	RCT	May 2007 with a follow-up conducted in Oct 2010	12 months	70.4±11.5/69.7±12.6	135/131	Inpatient and outpatient	Telephone	Readmission (six months), MLHFQ (six months), mortality (12 months)
Barzou et al. 2014 [16]	Iran	RCT	2013	Not reported	Not reported	32/32	Hospitalization	Face to face	Ferrans and Powers’ quality-of-life index (one month)
Riegel et al. 2004 [18]	USA	RCT	1999-2001	12 months	72.95±12.97/72.64±12.0	31/29	Hospitalization	Telephone and face to face	Readmission (three months) SCHFI (three months)

The mean age of the patients with heart failure ranged from 69.7 to 72.5 years. Two studies encompassed inpatients exclusively, while one study incorporated both inpatients and outpatients. Peer support was provided by telephone, face-to-face support, or a combination of telephone and face-to-face support.

**Figure 2:**
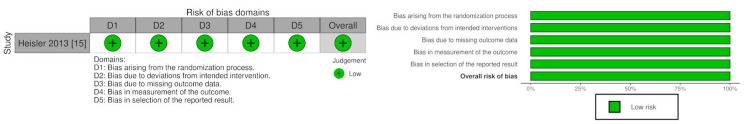
Risk of bias summary and graph for mortality

We used the risk-of-bias tool 2 to assess each outcome. We judged that mortality was rated as a “low” overall risk of bias (Figure 2).

**Figure 3:**
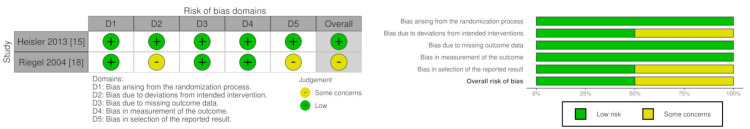
Risk of bias summary and graph for readmission

We judged that the readmission rate was rated as a “some concerns” overall risk of bias. This was because the measurement of deviations and outcomes was assessed as having “some concerns” (Figure 3).

**Figure 4:**
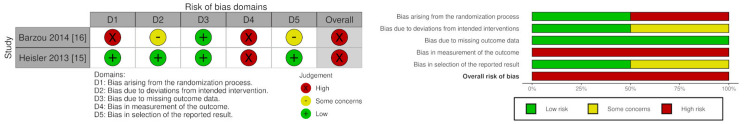
Risk of bias summary and graph for QoL

We judged that the QoL was rated as a “high” overall risk of bias. This was because the measurement of the randomization process and outcomes was assessed as having “high” (Figure 4).

**Figure 5:**
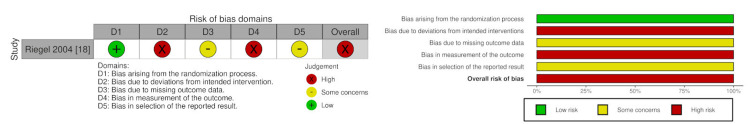
Risk of bias summary and graph for the SCHFI

We judged that the SCHFI was rated as a “high” overall risk of bias. This was because the measurement deviations and outcomes were assessed as having “high” (Figure 5).

Table 2 summarizes the SoFs in this review.

**Table 2:** Summary of the findings for the comparison of indicated outcomes between control and intervention a. Downgraded one level for serious limitations in the study design (randomized process and measurement of the outcome). b. Downgraded two levels for very serious imprecision 95% CI=40.04-1.64 including null effects. c. Downgraded one level for serious limitations in the study design (intended intervention and measurement of the outcome). d. Downgraded one level for serious inconsistency. e. Downgraded two levels for a sample size that is small and does not meet OIS. f. Downgraded two levels for very serious imprecision 95% CI=0.61-2.21 including null effects. g. Downgraded two levels for serious imprecision 95% CI=0.74-1.17 including null effects.

Summary of findings					
Outcomes	Anticipated absolute effects^*^ (95% CI)		Relative effect (95% CI)	No of participants (studies)	Certainty of the evidence (GRADE)
	Risk with control	Risk with intervention			
SCHFI	The mean SCHFI was 0	MD 19.2 lower (40.04 lower to 1.64 higher)	-	53 (1 RCT)	⨁◯◯◯ Very low^a,b^
QoL	-	SMD 2.03 higher (1.79 lower to 5.84 higher)	-	331 (2 RCTs)	⨁◯◯◯ Very low^c,d,e^
Mortality	115 per 1,000	133 per 1,000 (70 to 253)	RR 1.16 (0.61-2.21)	266 (1 RCT)	⨁⨁◯◯ Low^f^
Readmission	460 per 1,000	428 per 1,000 (340 to 538)	RR 0.93 (0.74-1.17)	354 (2 RCTs)	⨁⨁◯◯ Low^g^
*The risk in the intervention group (and its 95% confidence interval) is based on the assumed risk in the comparison group and the relative effect of the intervention (and its 95% CI).CI: confidence interval; MD: mean difference; RR: risk ratio; SMD: standardized mean difference					
GRADE Working Group grades of evidence High certainty: we are very confident that the true effect lies close to that of the estimate of the effect. Moderate certainty: we are moderately confident in the effect estimate: the true effect is likely to be close to the estimate of the effect, but there is a possibility that it is substantially different. Low certainty: our confidence in the effect estimate is limited: the true effect may be substantially different from the estimate of the effect. Very low certainty: we have very little confidence in the effect estimate: the true effect is likely to be substantially different from the estimate of effect. Compared to control of the intervention with peer support in heart failure Patient or population: heart failure Intervention: peer support Comparison: control					

**Figure 6:**
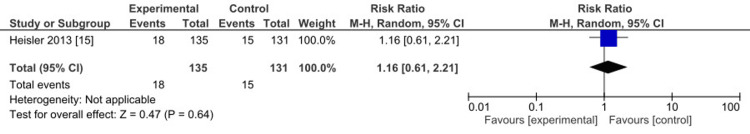
Forest plot for mortality

Peer support may have resulted in a slight increase in mortality compared with the control in patients with heart failure (one study, n= 266; RR=1.16, 95% CI=0.61-2.21, low certainty of the evidence) (Figure 6).

**Figure 7:**
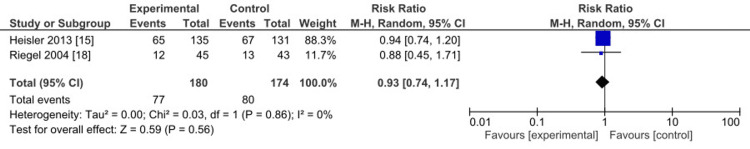
Forest plot for readmission

Peer support may have resulted in a reduction in the readmission rate of patients with heart failure compared with the control (two studies, n=331, RR=0.93, 95%CI=0.74-1.17, low certainty of the evidence) (Figure 7).

**Figure 8:**
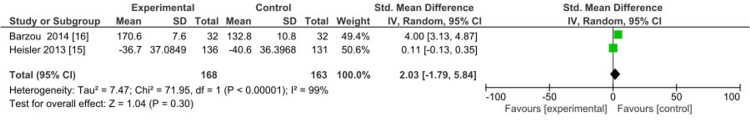
Forest plot for QoL

The evidence was very uncertain about the effect of peer support on QoL in patients with heart failure (two studies, n=231, SMD2.03 higher, 95%CI=1.79 lower to 5.84 higher, very low certainty of the evidence) (Figure 8).

**Figure 9:**
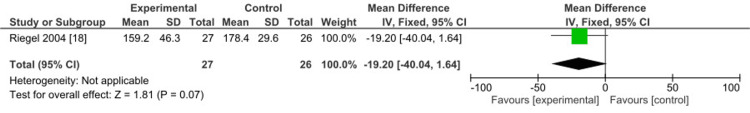
Forest plot for SCHFI

The evidence was very uncertain about the effect of peer support on SCHFI in patients with heart failure (one study (n= 53); MD19.2 lower, 95%CI=40.04 lower to 1.64 higher; very low certainty of the evidence) (Figure 9). No study reported the time to readmission.

Discussion

In this review, we found that peer support may result in a slight increase in mortality. Peer support may result in a slightly reduced readmission rate in patients with heart failure. Peer support for patients with heart failure may have little to no effect on QoL and self-efficacy, but the evidence is very uncertain.

This review suggests that peer support may increase mortality in patients with heart failure compared to control or nurse-led education. This result is inconsistent with the effectiveness of peer support in improving HbA1c for patients with DM [25]. In DM, self-management goals are specific and easily measurable, such as improving blood sugar control and HbA1c, while, in heart failure, self-management is a means of preventing exacerbations, which can make it difficult for patients to control themselves [15]. Heart failure is delineated according to the New York Heart Association functional classification, contingent upon the severity of symptoms and physical activity [4]. In this systematic review, health is defined as an absence of symptoms of heart failure and unrestricted physical activity. The influence of peer support on the QoL of patients with heart failure is uncertain. Peer support may function when patients are healthy enough to participate meaningfully and perceive that the more they participate, the more likely they are to improve their symptoms and QoL [15]. In one of the included studies, 82% of participants reported that peer support was provided less than 50% of the target number of times during the intervention period [15]. It is possible that the patients with heart failure were in poor health and were unable to access peer support and that, as a result, the peer support may not have been effective. Other reasons may be that our systematic review included both heart failure with preserved EF (HFpEF) and HFrEF. A prior RCT showed that non-pharmacologic treatment of disease management reduces mortality in HFrEF [26]. However, the effectiveness of the disease management program is not clear in HFpEF [27]. In the included studies of review, ejection fraction (EF) was not reported in two studies [15,16], while another study amalgamated both HFpEF and HFrEF, precluding separate analyses [19]. It is possible that HFpEF is a complex disease with a multifaceted phenotype that makes disease management difficult with patient-to-patient peer support.

There is conflicting evidence about the efficacy of peer support [28]. Peer support is frequently utilized in mental disorders, with the World Health Organization advocating its incorporation within mental health [29]. For instance, Israel has instituted a diverse assortment of government-endorsed peer services, deemed as indispensable resources within the mental health landscape [30]. Furthermore, peer support has been promoted as a key strategy for diabetes self-management [31]. However, peer support was not effective in improving the QoL of the patients with HIV [32]. Our findings add to evidence suggesting that the effectiveness of peer support for patients with heart failure is uncertain. Peer support for patients with heart disease does not work just by sharing experiences [33]. Studies incorporating patient education spearheaded by medical professionals, alongside discussions between these professionals and patients with heart failure, have been associated with reductions in mortality and readmissions up to seven months post-intervention [34]. For patients contending with heart failure, peer support may incorporate a significant component of specialized medical comprehension. It is not clear how peer support produces results [35]. Despite these hurdles, peer support is gradually garnering recognition as a pivotal facet of disease management, prompting concerted efforts to broaden its application. It is prudent to acknowledge that the effectiveness of peer support varies across different diseases, thus necessitating decisions grounded in evidence-based judgment.

Peer support can be harmful in some circumstances. For instance, it has been observed that peer support could escalate smoking rates after three months in teens with a history of delinquency [36]. Some sites include messages suggesting or encouraging unhealthy behaviors, such as suicide methods, HIV transmission methods, and insulin use for weight loss [37]. Online peer support may expose individuals to harsh comments, slurs, and cyberbullying [38]. This stems from the fact that the online self, unlike the offline self, can shirk responsibility for hostile or deviant actions [39]. For young patients contending with cancer, peer support on social media platforms may intensify feelings of disheartenment in response to distressing treatment narratives and survival information shared by others [40]. Informal peer supporters may inadvertently disseminate erroneous information [41]. Patients with heart failure are implored to adopt self-care practices, such as smoking cessation, moderate alcohol consumption, symptom self-monitoring adequate fluid and salt intake, exercise, healthy diet, and adherence to medication regimens [42]. However, peer support may exacerbate unhealthy behaviors, such as neglect of these recommended practices in patients with heart failure. While the exact mechanism remains elusive, our meta-analysis suggested peer support may increase mortality for patients with heart failure. It is of paramount importance to consider the potential benefits and detriments of peer support while making decisions based on need and preference according to the disease.

To the best of our knowledge, this systematic review constitutes the first study to appraise critical outcomes, such as mortality and readmission rates, in relation to peer support for patients with heart failure. In addition, we employed a robust methodological strategy that incorporated an exhaustive literature search and a predetermined protocol (https://osf.io/uxgam/) for analysis [17,43]. However, this review has some potential limitations. First, our review included only three RCTs, and the results exhibited low or very low certainty of evidence. Several studies did not include EF or brain natriuretic peptide, both measures of heart failure severity. In addition, one study had less than 50% participation in peer support. Comprehensive multidisciplinary management is recommended for patients with heart failure and may have included cases in which peer support for heart failure was not indicated. The certainty of the evidence for peer support for patients with heart failure may have been low or very low due to the severity of heart failure and low participation rates. Although our results suggest that peer support for patients with heart failure may not be ripe for implementation within clinical practice, future well-designed RCTs could potentially alter this inference. Second, we could not perform subgroup analyses and sensitivity analyses due to the limited data and were unable to evaluate heterogeneity and robustness. These issues should also be investigated in future works.

## Conclusions

Peer support for patients with heart failure may result in a slight increase in mortality. Peer support may result in a slightly reduced readmission rate in patients with heart failure. Peer support for patients with heart failure may have little to no effect on QoL and self-efficacy, but the evidence is very uncertain.

At this point, peer support for patients with heart failure may not be recommended. The evidence for peer support for heart failure may be low or very low due to the inclusion of only three RCTs in this study, the unspecified severity of heart failure among participants, and the high deviation rate. Additional RCTs are needed to confirm our conclusions and elucidate the mortality and readmission rate of peer support for patients with heart failure. Additionally, the study analyzed here did not incorporate a time-to-readmission outcome. Future RCTs should consider including time to readmission as an evaluative metric.

## Appendices

**Table 3:** MEDLINE (PubMed) search strategy

#1	“Heart failure” [mh]
#2	“Cardiac Failure” [tiab]
#3	“cardiac insufficiency” [tiab]
#4	“CHF” [tiab]
#5	“HFrEF” [tiab]
#6	“HFpEF” [tiab]
#7	“Heart Decompensation” [tiab]
#8	“Heart Failure” [tiab]
#9	“left ventricular failure” [tiab]
#10	“LVEF” [tiab]
#11	“Myocardial Failure” [tiab]
#12	#1 OR #2 OR #3 OR #4 OR #5 OR #6 OR #7 OR #8 OR #9 OR #10 OR #11
#13	“social support” [mh]
#14	“Self Help Groups” [mh]
#15	“peer group” [mh]
#16	“Self-Management” [mh]
#17	“peer” [tiab]
#18	“buddy” [tiab]
#19	“buddies” [tiab]
#20	“mutual” [tiab]
#21	#13 OR #14 OR #15 OR #16 OR #17 OR #18 OR #19 OR #20
#22	“support” [tiab]
#23	“communication” [tiab]
#24	#22 OR #23
#25	“self-help” [tiab]
#26	“patient education” [tiab]
#27	“health class” [tiab]
#28	“health educational” [tiab]
#29	“health training” [tiab]
#30	“medical training” [tiab]
#31	#25 OR #26 OR #27 OR #28 OR #29 OR #30
#32	#21 AND #24 OR #31
#33	randomized controlled trial [pt]
#34	controlled clinical trial [pt]
#35	randomized [tiab]
#36	placebo [tiab]
#37	drug therapy [sh]
#38	randomly [tiab]
#39	OR [tiab]
#40	#33 OR #34 OR #35 OR #36 OR #37 OR #38 OR #39
#41	animals [mh]
#42	humans [mh]
#43	#41 NOT #42
#44	#40 NOT #43
#45	#12 AND #32 AND #44

**Table 4:** CENTRAL (Cochrane Library) search strategy

#1	[mh "Heart failure"]
#2	"Cardiac Failure":ti,ab
#3	"cardiac insufficiency":ti,ab
#4	CHF:ti,ab
#5	HFrEF:ti,ab
#6	HFpEF:ti,ab
#7	"Heart Decompensation":ti,ab
#8	"Heart Failure":ti,ab
#9	"left ventricular failure":ti,ab
#10	LVEF:ti,ab
#11	"Myocardial Failure":ti,ab
#12	#1 OR #2 OR #3 OR #4 OR #5 OR #6 OR #7 OR #8 OR #9 OR #10 OR #11
#13	[mh "social support"]
#14	[mh "Self Help Groups"]
#15	[mh "peer group"]
#16	[mh Self-Management]
#17	#13 OR #14 OR #15 OR #16
#18	peer:ti,ab
#19	buddy:ti,ab
#20	buddies:ti,ab
#21	mutual:ti,ab
#22	#18 OR #19 OR #20 OR #21
#23	support:ti,ab
#24	communication:ti,ab
#25	#23 OR #24
#26	"self-help":ti,ab
#27	"patient education":ti,ab
#28	"health class":ti,ab
#29	"health educational":ti,ab
#30	"health training":ti,ab
#31	"medical training":ti,ab
#32	#25 OR #26 OR #27 OR #28 OR #29 OR #30
#33	#12 AND #17 OR #22 AND #25 OR #32

**Table 5:** EMBASE (Dialog) search strategy

S1	EMB.EXACT.EXPLODE("heart failure")
S2	AB("cardiac failure") OR TI("cardiac failure")
S3	AB("cardiac insufficiency") OR TI("cardiac insufficiency")
S4	AB("chf") OR TI("chf")
S5	AB("hfref") OR TI("hfref")
S6	AB("hfpef") OR TI("hfpef")
S7	AB("heart decompensation") OR TI("heart decompensation")
S8	AB("heart failure") OR TI("heart failure")
S9	AB("left ventricular failure") OR TI("left ventricular failure")
S10	AB("lvef") OR TI("lvef")
S11	AB("myocardial failure") OR TI("myocardial failure")
S12	S1 OR S2 OR S3 OR S4 OR S5 OR S6 OR S7 OR S8 OR S9 OR S10 OR S11
S13	EMB.EXACT.EXPLODE("social support")
S14	EMB.EXACT.EXPLODE("self help groups")
S15	EMB.EXACT.EXPLODE("peer group")
S16	EMB.EXACT.EXPLODE("self-management")
S17	AB("peer") OR TI("peer")
S18	AB("budy") OR TI("budy")
S19	AB("budies") OR TI("budies")
S20	AB("mutual") OR TI("mutual")
S21	S17 OR S18 OR S19 OR S20
S22	AB("support") OR TI("support")
S23	AB("communication") OR TI("communication")
S24	S22 OR S23
S25	S21 AND S24
S26	AB("self help") OR TI("self help")
S27	AB("patient education") OR TI("patient education")
S28	AB("health class") OR TI("health class")
S29	AB("health educational") OR TI("health educational")
S30	AB("health training") OR TI("health training")
S31	AB("medical training") OR TI("medical training")
S32	S13 OR S14 OR S15 OR S16 OR S25 OR S27 OR S28 OR S29 OR S30 OR S31
S33	(ab(random*) OR ti(random*)) OR (ab(clinical NEAR/1 trial*) OR ti(clinical NEAR/1 trial*)) OR (EMB.EXACT("health care quality"))
S34	S12 AND S32 AND S33

**Table 6:** ICTRP search strategy, advanced search

#1	Conditions (heart failure OR cardiac failure OR cardiac insufficiency OR HFrEF OR HFpEF OR heart decompensation OR LVEF OR myocardial failure)
#2	Intervention (social support OR Self-Help Groups OR peer group OR Self-Management OR patient education OR health educational OR health training OR buddy support OR mutual support)

**Table 7:** ClinicalTrials.gov search strategy, advanced search

#1	Heart failure OR cardiac failure OR cardiac insufficiency OR CHF OR HFrEF OR HFpEF OR heart decompensation OR heart failure OR left ventricular failure OR LVEF OR myocardial failure
#2	social support OR Self-Help Groups OR peer group OR Self-Management OR peer support OR peer communication OR buddy support OR buddy communication OR buddies support buddies communication OR mutual support OR mutual communication OR self-help OR patient education OR health class OR health educational OR health training OR medical training
#3	#1 AND #2
